# Systematic heterogeneity and prognostic significance of cell proliferation in colorectal cancer.

**DOI:** 10.1038/bjc.1998.152

**Published:** 1998-03

**Authors:** R. Palmqvist, A. Oberg, C. BergstrÃ¶m, J. N. RutegÃ¥rd, B. Zackrisson, R. Stenling

**Affiliations:** Department of Pathology, UmeÃ¥ University, Sweden.

## Abstract

**Images:**


British Journal of Cancer (1998) 77(6), 917–925
© 1998 Cancer Research Campaign

### Systematic heterogeneity and prognostic significance of cell proliferation in colorectal cancer

**R Palmqvist[1], Å Öberg[2], C Bergström[1,3], JN Rutegård[2,4], B Zackrisson[3] and R Stenling[1]**

Departments of [1]Pathology, [2]Surgery and [3]Oncology, Umeå University, 901 87 Umeå, Sweden; [4]Department of Surgery, District Hospital of Örnsköldsvik, 891 89 Örnsköldsvik, Sweden

**Summary** The prognosis of colorectal cancer has not significantly changed during the last 30 years. While evaluation of tumour cell proliferation may provide prognostic information, results obtained so far have been contradictory. Heterogeneity in tumour cell proliferation may explain these contradictions. With in vivo injection of iododeoxyuridine (IdUrd), estimation of labelling index (LI), S-phase transit time ($T_s$) and potential doubling time ($T_{pot}$) may be performed from a single sample. A total of 109 colorectal cancers were studied after in vivo injection of IdUrd before surgical removal. From each cancer, four to eight samples were processed for both flow cytometrical (FCM) and immunohistochemical (IHC) visualization of IdUrd incorporation. LI/IHC was morphometrically quantified at both the luminal border and the invasive margin of these tumours. LI was significantly higher at the luminal border compared with the invasive margin, although they were correlated with each other. Using combined IHC and FCM methods, rapidly growing colorectal cancers (high LI and/or low $T_{pot}$) showed an increased survival (significant for LI at the invasive margin and for $T_{pot}$ at both the invasive margin and the luminal border) in the entire unselected material and for radically removed Dukes' B tumours. FCM data alone did not discriminate for survival, with the exception of $T_s$ in diploid and radically removed Dukes' B tumours.

**Keywords:** cell proliferation; iododeoxyuridine; heterogeneity; immunohistochemistry; flow cytometry; colorectal carcinoma

Colorectal cancer is the second leading cause of cancer death in most countries with a western type of diet. Treatment is still based on surgical removal of the tumours, and prognosis has not changed much during the last 30 years; approximately 50% of patients with colorectal cancer die from their disease. Nevertheless, clinical trials in recent years have shown that both adjuvant chemotherapy (IMPACT investigators, 1995; Moertel et al, 1995) and treatment with monoclonal antibodies (Riethmuller et al, 1994) might improve the survival in patients with Dukes' C colorectal cancer. In addition, other studies have shown that the surgical technique is important for prognosis (MacFarlane et al, 1993) and that preoperative radiotherapy reduces the risk of local recurrence (Holm et al, 1994).

So far, Dukes' classification of tumour stage is the most important prognostic factor and few other tissue derived alterations that correlate with the clinical outcome of colorectal cancer exist. Identification of specific, independent biological parameters correlating with biological tumour behaviour could in the future guide clinicians in selecting patients for both chemo- and radiotherapy.

Malignant tumours with a rapid cell proliferation may be candidates for treatment with altered radiotherapy schedules (Dische and Saunders, 1989) and, for similar reasons, they may be more susceptible to chemotherapy (Riccardi et al, 1991). In this respect, several factors, such as S-phase (Bauer et al, 1993), proliferative cell nuclear antigen (PCNA) (Al-Sheneber et al, 1993; Teixeira et al, 1994) and Ki-67 (Kubota et al, 1992; Baretton et al, 1996) are of interest. The results obtained so far are contradictory for colorectal cancer. There are, however, indications that rapid tumour cell proliferation might be correlated with poorer prognosis (Bauer et al, 1987; Harlow et al, 1991).

Multiple parameters associated with tumour cell proliferation can be measured by in vivo injection of bromo- (BrdUrd) or iododeoxyuridine (IdUrd), and dual-parameter flow cytometric analysis of a single sample (Begg et al, 1985). This technique allows determination of labelling index (LI), the duration of S-phase ($T_s$) and potential doubling time ($T_{pot}$) in colorectal cancer (Rew et al, 1991; Wilson et al, 1993*a* and *b*; Terry et al, 1995). LI can be evaluated either by FCM of cell suspensions or by morphometry in immunohistochemically (IHC) stained tissue sections. $T_{pot}$ can thus be calculated with LI derived either from histology or from FCM (Bennett et al, 1992). The findings by Dische and Saunders (1989), Rew et al (1991) and Wilson (1991) suggest that cell proliferation evaluated with this technique might be of independent prognostic value.

Colorectal cancer show a marked intratumoral heterogeneity with respect to several parameters, such as DNA content (Koha et al, 1990) and histological differentiation (Jass et al, 1986), and to proliferative parameters, such as S-phase fraction (Lindmark et al, 1991), PCNA (Teixeira et al, 1994) and expression of Ki-67 (Shepherd et al, 1988). Wilson et al (1993*b*) used flow cytometry alone to measure $T_{pot}$, and have also described intratumoral heterogeneity in colorectal cancers. In addition, Taniyama et al (1993) have reported that mainly moderately differentiated colorectal cancer can be poorly differentiated at the invasive margin and that such tumour regions can also differ with respect to proliferative activity and tumour spread.

The aim of this study was (1) to evaluate whether there is a systematic heterogeneity with respect to cell proliferation between deep (invasive margin) and superficial (luminal border) parts of colorectal cancers and (2) to investigate the prognostic impact of LI, $T_s$ and $T_{pot}$.

*Received 17 February 1997*
*Revised 24 June 1997*
*Accepted 27 June 1997*

*Correspondence to:* R Stenling

### MATERIAL AND METHODS

### Patients

One hundred and nine patients with colorectal cancer who underwent surgery at the University Hospital of Umeå (71 patients) and at the district hospitals of Örnsköldsvik (13), Lycksele (9), Piteå (10) and Luleå (6) within the northern healthcare region of Sweden were prospectively included in the study. After hospital ethical committee approval, informed consent was obtained from the patients. Intravenous infusion of a single dose of 100 mg of IdUrd dissolved in 100 ml of 0.9% saline was given at a median of 5.2 h (range 1.1–8.9 h) before surgery. At the University Hospital of Umeå, fresh unfixed tumours were transported immediately after removal to the Department of Clinical Pathology for further processing. Each tumour was systematically sampled by dividing the tumour into equal numbers of central and peripheral regions, with at least four regions in total. From each of these regions, adjacent samples were taken for routine histological evaluation, morphometric analysis of LI in IHC-stained tissue sections and for flow cytometric analysis of cell kinetic relations, i.e. LI, $T_s$ and $T_{pot}$ (Figure 1). At the district hospitals, one central and one peripheral tumour sample was taken for flow cytometric analysis. However, for routine histological evaluation and morphometric analysis of LI in tissue sections, a minimum of four samples were systematically collected from both central and peripheral tumour regions.

Of the 109 patients with colorectal cancer included in the study, 53 were men and 56 were women. Twenty-five tumours were classified as Dukes' stage A, 45 as Dukes' B and 39 as Dukes' C. Ninety out of 109 tumours were classified as radically removed, of which 25 were Dukes' stage A, 39 Dukes' B and 26 Dukes' C. Colorectal cancer primary sites were distributed with an expected frequency, i.e. 37 rectal, 26 sigmoid and 21 caecal cancers. The remaining 24 tumours were located in the ascending, transverse and descending colon.

All follow-up data were obtained by ÅÖ. The follow-up time of living patients ranged from 18 to 78 months, with a median of 37 months. To verify the accuracy of cancer-specific survival used in this study, curves of cancer-specific survival for the entire material ($n$ = 109) were compared with relative survival data, i.e. death rates of the colorectal cancer group compared with death rates of a corresponding normal population. The two survival curves showed good agreement. In addition, the survival curve for the group of patients treated at the district hospitals corresponded well with the survival curve from patients treated at the University Hospital of Umeå.

### Flow cytometric analysis of IdUrd nuclear incorporation

The rod-shaped tissue samples of size 3–5 × 3–5 × 10–20 mm, taken for FCM analyses from each colorectal carcinoma, were immediately fixed in 70% ethanol and then stored at 4°C until analysis. Before FCM processing, one piece of tissue from each of these specimens was minced into smaller pieces, subsequently digested using 0.4 mg ml$^{-1}$ pepsin (Sigma Chemical, St Louis, MO, USA) in 0.1 M hydrochloric acid at 37°C for 30 min during continuous agitation and filtered through a 50-μm nylon mesh (Nylander et al, 1994). The concentration of nuclei was adjusted to $2 \times 10^6$ ml$^{-1}$ and the solution centrifuged for 5 min at 2000 r.p.m. to sediment the nuclei. The resulting nuclear pellet was partly denatured in 2 ml of 2 M hydrochloric acid for 15 min. The denaturation process was stopped by addition of 2.5 ml of borax (JT Baker Chemical, Phillipsburg, NJ, USA) in 2.5 ml of phosphate-buffered saline (PBS) added with 0.5% Tween 20 (Sigma Chemical). After a wash in PBS, the cell suspension was incubated at room temperature for 30 min with a BrdUrd/IdUrd monoclonal antibody diluted 1:20 (Dakopatts, Denmark), washed in PBS and incubated with fluorescein isothiocyanate- (FITC) conjugated rabbit anti-mouse F(ab)$_2$ fragment (F313, Dako) (1:50 dilution) for 30 min at room temperature. After a final wash in PBS, the pellet was resuspended in a propidium iodide solution containing RNAase and left in the dark at 4°C for at least 30 min before analysis. The suspensions were analysed on a FACScan flow cytometer (Becton Dickinson) using the FACScan or LYSYS software (Becton Dickinson). Debris, damaged cells and doublets were excluded by gating on a forward- and side-scatter dot plot and on the FL2 width and FL2 area dot plot. The analysis was performed so that the $G_1$ diploid peak was situated near channel 200, with a tetraploid peak around 400. Normal colorectal mucosa specimens from each patient were used as an internal control. With ethanol-fixed material, as used in this study, multipeak profiles were rare, and ploidy analysis using special software algorithms was not necessary. From each specimen, 10 000 cells or more were analysed for $T_s$, LI/FCM and $T_{pot}$/FCM. LI/FCM and $T_{pot}$/FCM were calculated as suggested by Steel (1977), when λ was assumed to be 0.8 according to Wilson and McNally (1992). Correction of LI/FCM for mitotic division from time of infusion with IdUrd and to time for fixation was made according to a technique described by Lochrin et al (1992). According to the DNA histogram calculation, the DNA index was determined and, if one or more samples from the same tumour were found to be aneploid, that tumour was classified as aneuploid. In aneuploid tumours, LI/FCM analyses was restricted to the aneuploid cell population. The CV (coefficient of variation) of the DNA peaks for diploid cells in aneuploid tumours had a mean value of 6.3 (range 2.8–10.6). The DNA peaks of aneuploid tumour cells had a mean CV of 7.1 (2.9–12.4).

### Immunohistochemical procedures

For histopathological analysis, a tumour sample cut perpendicular to the mucosal surface was collected from each region (see above), fixed overnight in 10% formalin buffered to neutrality with phosphate buffer and subsequently embedded in paraffin. The sample included a section from the entire tumour, i.e. from the intestinal lumen all the way to the submucosa, muscular layer or to the

**Figure 1** Schematic drawing that shows the sampling scheme used when taking four pieces of tissue from one colorectal cancer

© Cancer Research Campaign 1998

**Figure 2** Schematic cross-section of the large bowel showing the luminal border and the invasive margin tumour compartments analysed. Luminal border is defined as the most superficial fourth of the tumour depth within the bowel wall. Invasive margin is defined as the deepest fourth peripheral fat. Four-micrometre sections were cut from the paraffin blocks (four from each tumour) and left to dry overnight at 37°C (Nylander et al, 1994), followed by 30 min at 56°C. After dewaxing and rehydration, the slides were digested with 0.4% pepsin in 0.01 M hydrochloric acid at 37°C for 30 min, followed by 2 M hydrochloric acid treatment at room temperature for 20 min. After rinsing in PBS, the slides were stained in an automated immunostainer (Ventana ES, Ventana, Tucson, AZ, USA) using an anti-IdUrd/BrdUrd monoclonal antibody (BrdU, Becton Dickinson) at a dilution of 1:600. Antibody visualization was performed according to the Ventana program. The slides were then manually counterstained with Mayer's haematoxylin for 1.5 min. The Ventana ES is constrained to perform all incubations at a 40°C slide reaction temperature.

### Morphometrical analysis

One IHC-stained section from each of the four tissue samples (collected as described above) was used for morphometric analysis. A Zeiss microscope (63 × objective magnification) equipped with a square lattice in the eyepiece was used to define the proper size of the counting frame in each of the tumours evaluated (Gundersen et al, 1988). From each tumour about 40 fields of vision were measured in a systematic random fashion, so that the first field was selected at random while subsequent fields were chosen systematically by adjusting the distance between individual fields of vision roughly proportional to the overall area in question.

Two tumour compartments were regularly evaluated in each section. One was represented by the most superficial fourth (corresponding to the luminal tumour part), while the other was represented by the deepest fourth (corresponding to the invasive margin) (Figure 2). The two compartments were analysed separately without prior knowledge of the results from earlier morphometrical evaluations.

Two unbiased counting frames (Gundersen, 1977) were chosen within each of the 40 fields of vision. One of these frames was used to count positive nuclei and the other to count negative nuclei. These counts were then used to calculate the numerical density of positive and negative nuclei. The labelling index was calculated as:

$$\text{LI/IHC} = \frac{Q_p/A_p}{(Q_p/A_p) + (Q_n/A_n)} \qquad \text{(equation 1)}$$

where $Q_p$ is the number of positive nuclei, $A_p$ is the area of the counting frame used to count positive nuclei, $Q_n$ is the number of negative nuclei and $A_n$ is the frame area used for counting the negative nuclei. Only epithelial tumour nuclei were examined.

For each tumour, about 200 positive and 200 negative nuclei were counted from both the most superficial (luminal border) and the deepest (invasive margin) fourths. In practice, the intratumoral coefficient of error (CE) of the numerical density for positive nuclei for each defined tumour compartment (e.g. invasive margin) was between 0.05 and 0.15. The technique of Lochrin et al (1992) was used to correct LI for mitotic division from time of infusion with IdUrd to time of fixation.

With these procedures, LI was quantitatively evaluated as a mean value for the entire tumour, using FCM analysis of nuclear suspensions, and as an index for both luminal border (most superficial fourth) and invasive margin (deepest fourth) in immunohistochemically stained sections from each tumour. $T_{pot}$/IHC was calculated according to Bennet et al (1992), using LI from either the invasive margin or the luminal border:

$$T_{pot}\text{/IHC} = \frac{\lambda \times T_s}{\text{LI/IHC}} \qquad \text{(equation 2)}$$

Reliability of quantitative results from morphometrical methods (Gundersen and Osterby, 1981; Gundersen and Jensen, 1987) depends on uniform sampling at all levels from a random systematic approach, however, in clinicopathological practice, such a procedure is not always possible to achieve. In this study, all sampling steps were performed in a uniform way, with the exception of the first step when a strict systematic sampling was used. We therefore tested our sampling error in measuring LI/IHC using four tumours that were sampled according to a strictly systematic scheme and to a morphometrically correct random systematic scheme. The comparison between the two sampling procedures showed only a minor sampling error, with a CE for the difference between the paired tumour samples of 0.04.

### Statistics

Spearmans correlation coefficient ($r$) and Wilcoxon matched-pairs signed-rank test were used to compare sets of parameters measured on the same tumour. To compare distributions of a variable for groups, the Kruskal–Wallis test was used. For morphometrical analysis, CE was calculated according to methods described by West et al (1990). Kaplan–Meier's method was used to estimate the cancer-specific survival, and comparison between groups was performed using the log-rank test. Death with known locoregional or distant metastases was processed as an event and, if no event occurred, the patient was censored at the time of the last clinical follow-up. A $P$-value less than 0.05 was considered to be statistically significant. The median value or the highest/lowest quartiles of the entire patient material were used as cut-off points to create groups for comparison of different parameters. Statistical analyses were performed using SPSS version 6.1.3 (SPSS, IL, USA).

### RESULTS

Of the 109 colorectal cancers, 89 could be evaluated with flow cytometry while 97 were analysed morphometrically. Of these, the invasive margin could be evaluated in 97 and the luminal border in 94 of the cancers. All tumours in which light microscopic shortcomings prevented identification of the luminal border and/or the invasive margin, and/or measurement of the distance between the luminal border and the invasive margin were excluded. At least

© Cancer Research Campaign 1998

**Figure 3** Paired observations of LI/IHC both at the luminal border and at the invasive margin. LI/IHC was significantly higher at luminal border ($P < 0.001$). Only nine tumours had higher LI at the invasive margin compared with the luminal border two tissue samples from each tumour had to be measurable for a tumour to be included in the morphometrical study. Tumours studied by flow cytometry alone, or by combined immunohistochemically and flow cytometrically evaluated $T_{pot}$, were also excluded if technical problems prevented accurate recording of the timespan between infusion of IdUrd and fixation of the tumour or production of adequate DNA profiles and/or dot plot diagrams.

### LI

LI/IHC measured morphometrically was significantly higher at the luminal border compared with the invasive margin ($P < 0.001$) (Figures 3 and 4). Only nine tumours showed higher LI at the invasive margin. Nevertheless, LI/IHC at the luminal border and the invasive margin were correlated to each other ($r = 0.66$, $P < 0.001$; Figure 5). No significant correlations were found for LI/IHC and Dukes' stage, grade or topography. Furthermore, no significant difference was observed between central and peripheral samples regarding LI/FCM, either for $T_s$ or for $T_{pot}$/FCM ($P = 0.3$, $P = 0.8$ and $P = 0.7$ respectively).

As expected, stage according to Dukes was a strong prognostic indicator $P < 0.001$ (Figure 6). For the entire unselected tumour material, colorectal cancers with low LI/IHC (below median value) at the invasive margin tended to have poorer prognosis than those with high LI/IHC. Tumours with very low LI/IHC at the invasive margin (lowest quartile used as cut-off level) showed significantly lower survivals ($P = 0.02$, Figure 7A). In radically removed tumours, very low LI/IHC (lower quartile as cut-off level) was associated with significantly lower survival rates than those with higher LI/IHC. These results were generally valid for all radically removed tumours ($P = 0.01$) and for radically removed Dukes' B tumours ($P = 0.01$), although a small number of tumours with low LI/IHC were recorded in the Dukes' B group (Figure 7B). No differences in survival were seen in the Dukes' C group. The survival pattern for LI/IHC at the luminal border was similar to that observed for the invasive margin but the differences were not statistically significant. LI measured with flow cytometry (LI/FCM) did not accurately predict survival either within the entire material or for radically removed Dukes' B tumours alone.

**Figure 4** Micrographs from two different compartments within the same colorectal adenocarcinoma immunohistochemically stained for iododeoxyuridine (IdUrd) showing (**A**) a higher fraction (LI) of labelled nuclei at luminal border compared with (**B**) the invasive margin

**Figure 5** Correlation between LI/IHC at luminal border and at invasive margin. LI was significantly correlated between the two regions ($r = 0.65$, $P < 0.001$)

*© Cancer Research Campaign 1998*

**Figure 6** Kaplan–Meier cancer-specific survival curves for Dukes' stages A (–), B (- - -) and C (– – –) in 109 colorectal carcinomas

**Figure 7** Kaplan–Meier survival curves for LI/IHC at the invasive margin of colorectal cancer. (**A**) Cancer-specific survival for the entire material ($P = 0.02$, $n = 97$). (**B**) Cancer-specific survival of radically removed Dukes' B tumours ($P = 0.01$, $n = 37$). - - -, LI above the lowest quartile; —, the lowest quartile

Radically removed tumours in which the difference in the magnitude of LI/IHC between the luminal border and the invasive margin was large tended to have a favourable prognosis, although this trend was not statistically significant ($P = 0.07$) when using the lowest quartile as cut-off level. The difference in LI/IHC between the luminal border and the invasive margin did not prognostically discriminate patients with radically removed Dukes' B tumours.

### $T_s$ and $T_{pot}$

$T_{pot}$/IHC at the invasive margin significantly predicted survival. Patients with very long $T_{pot}$/IHC at the invasive margin (highest quartile as cut-off level) had significantly lower survival rates both in the entire unselected tumour material ($P = 0.002$, Figure 8A) and in the group with radically removed Dukes' B colorectal cancers ($P < 0.001$, Figure 8B). $T_{pot}$/IHC at the invasive margin also discriminated for survival when the median value was used as cut-off point, although this was restricted to the group with radically removed Dukes' B colorectal cancers, and the significance level was lower ($P = 0.04$).

**Figure 8** Kaplan–Meier survival curves for potential doubling time ($T_{pot}$/IHC) at the invasive margin of colorectal cancer. (**A**) Cancer-specific survival for the entire material ($P = 0.002$, $n = 76$). (**B**) Cancer-specific survival of radically removed Dukes' B tumours ($P < 0.001$, $n = 32$). - - -, the highest quartile; —, $T_{pot}$ below the highest quartile

Using the highest quartile as the cut-off level, $T_{pot}$/IHC at the luminal border significantly predicted survival in the entire unselected material and for patients with radically removed Dukes'B tumours ($P = 0.008$ and $P = 0.002$ respectively). However, when the median value was used as the cut-off level, $T_{pot}$/IHC at the luminal border did not significantly predict survival.

$T_{pot}$ analysed by flow cytometry alone did not discriminate for survival, either generally or for radically removed Dukes' B tumours. A borderline $P$-value was recorded for the entire unselected material with the highest quartile as cut-off level ($P = 0.05$). No survival discrimination was found either for overall radically removed tumours or for radically removed Dukes' B tumours. $T_s$ did not discriminate for survival, with the exception of radically removed Dukes' B tumours, when the upper quartile was used as the cut-off level ($P = 0.04$).

### DNA ploidy

Thirty-nine of the 90 flow cytometrically evaluated tumours were diploid while the remaining 51 were classified as aneuploid. LI/FCM was significantly ($P = 0.001$) lower for diploid than for aneuploid colorectal cancers (Table 1). In contrast, significantly ($P = 0.04$) higher values of LI/IHC were observed at the invasive margin for diploid compared with aneuploid cancers. Diploid and aneuploid tumours did not differ for LI/IHC at the luminal border. The magnitudes of the calculated absolute differences between LI/IHC at the luminal border and at the invasive margin were significantly higher in aneuploid than in diploid tumours ($P = 0.001$, Table 1).

© Cancer Research Campaign 1998

**Table 1** Mean values for parameters associated with cell proliferation and clinicopathological characteristics in 109 colorectal cancers

| | *n* | LI | | | Diff. | $T_s$ | $T_{pot}$ | | |
|---|---|---|---|---|---|---|---|---|---|
| | | Inv (%) | Lum (%) | Fcm (%) | LI (%) | (h) | Inv (days) | Lum (days) | Fcm (days) |
| Sex | | | | | | | | | |
| Male | 53 | 9.1* | 14.6* | 10.3 | 5.3 | 8.1 | 4.6* | 2.9 | 3.7 |
| | | (7.2–10.9) | (12.2–17.1) | (8.2–12.4) | (3.7–7.0) | (6.9–9.3) | (3.2–6.0) | (1.9–3.9) | (3.0–4.5) |
| Female | 56 | 11.5 | 18.3 | 10.9 | 6.7 | 7.4 | 2.9 | 1.7 | 4.2 |
| | | (9.9–13.1) | (16.2–20.3) | (8.9–12.8) | (4.9–8.5) | (6.6–8.3) | (2.1–3.8) | (1.3–2.1) | (2.8–5.6) |
| Topography | | | | | | | | | |
| Right colon | 37 | 10.3 | 16.5 | 7.8*[a] | 5.6 | 7.1 | 3.1 | 2.2 | 4.0 |
| | | (8.3–12.2) | (13.3–19.8) | (6.4–9.2) | (2.8–8.3) | (6.1–8.1) | (2.1–4.1) | (1.4–3.0) | (3.0–4.9) |
| Left colon | 35 | 10.4 | 17.3 | 13.9 | 6.4 | 7.8 | 4.1 | 2.5 | 2.8 |
| | | (8.0–12.8) | (13.5–21.0) | (10.6–17.2) | (3.5–9.4) | (6.3–9.3) | (2.1–6.0) | (1.2–3.8) | (1.9–3.6) |
| Rectum | 37 | 10.4 | 17.1 | 11.6 | 6.6 | 8.5 | 3.9 | 2.0 | 4.7 |
| | | (8.2–12.5) | (14.5–19.8) | (8.9–14.2) | (4.7–8.4) | (7.0–9.9) | (2.6–5.3) | (1.3–2.6) | (2.9–6.8) |
| Stage (Dukes) | | | | | | | | | |
| A | 25 | 11.5 | 18.3 | 9.7 | 6.8 | 8.0 | 3.4 | 1.8 | 5.2 |
| | | (8.9–14.1) | (14.8–21.7) | (6.8–12.5) | (4.6–9.0) | (6.5–9.5) | (1.7–5.0) | (1.0–2.6) | (2.6–7.8) |
| B | 45 | 10.6 | 17.1 | 10.5 | 6.5 | 6.9 | 3.0 | 2.0 | 3.1 |
| | | (8.7–12.5) | (14.5–19.6) | (8.5–12.5) | (4.5–8.4) | (6.1–7.7) | (2.2–3.7) | (1.3–2.7) | (2.4–3.9) |
| C | 39 | 9.2 | 14.5 | 11.3 | 4.9 | 8.7 | 5.2 | 2.9 | 4.2 |
| | | (7.0–11.4) | (11.8–17.2) | (8.6–14.1) | (2.5–7.3) | (7.1–10.2) | (3.1–7.2) | (1.6–4.3) | (2.9–5.5) |
| Grade | | | | | | | | | |
| Well | 8 | 12.5 | 16.4 | 8.5 | 3.9 | 7.1 | 2.2 | 1.6 | 4.1 |
| | | (7.9–17.1) | (12.2–20.5) | (4.4–12.6) | (0.4–7.4) | (5.2–8.9) | (1.1–3.3) | (0.8–2.4) | (0.2–8.1) |
| Moderate | 90 | 10.3 | 16.6 | 10.7 | 6.1 | 7.9 | 3.8 | 2.3 | 4.1 |
| | | (8.9–11.6) | (14.7–18.4) | (9.1–12.2) | (4.8–7.5) | (7.1–8.7) | (2.9–4.8) | (1.7–2.9) | (3.2–5.0) |
| Poor | 11 | 9.5 | 16.3 | 11.7 | 6.8 | 6.8 | 3.7 | 1.8 | 2.5 |
| | | (4.5–14.5) | (10.4–22.3) | (6.2–17.2) | (0.7–13.0) | (4.6–9.0) | (1.0–6.5) | (0.4–3.1) | (0.7–4.3) |
| DNA ploidy | | | | | | | | | |
| Diploid | 39 | 12.2* | 16.4 | 7.9** | 4.0** | 6.8* | 3.0* | 2.2 | 4.4* |
| | | (10.0–14.4) | (13.7–19.1) | (6.5–9.2) | (2.1–5.9) | (6.1–7.5) | (2.0–4.0) | (1.4–2.9) | (3.3–5.4) |
| Aneuploid | 51 | 9.3 | 17.3 | 12.7 | 7.8 | 8.5 | 4.3 | 2.2 | 3.6 |
| | | (7.6–11.0) | (14.9–19.8) | (10.7–14.7) | (6.0–9.6) | (7.4–9.6) | (3.2–5.5) | (1.6–2.9) | (2.5–4.8) |

*n*, Number of colorectal cancers in each subgroup; LI/inv, labelling index at invasive margin; LI/lum, labelling index at luminal border; LI/fcm, labelling index measured with flow cytometry alone; Diff., the difference between LI at luminal border and invasive margin, $T_s$, S-phase time; $T_{pot}$/inv, potential doubling time measured with LI/IHC from the invasive margin; $T_{pot}$/lum, potential doubling time measured with LI/IHC from the luminal border. $T_{pot}$/fcm, potential doubling time measured with flow cytometry alone. *$P < 0.05$ between groups of the same proliferative-associated parameter. **$P < 0.01$ between groups of the same proliferative-associated parameter. Numbers within brackets represent 95% confidence intervals of the mean. [a]LI/fcm from right colon was significantly lower than values from both left colon and rectum.

Concerning survival, no prognostic information was seen for ploidy itself, either in the total unselected material ($P = 0.8$) or in the group of radically removed tumours ($P = 0.6$). With one exception (see below), the prediction of survival from parameters associated with cell proliferation (LI, $T_s$, $T_{pot}$), regardless of method of evaluation (IHC and/or FCM), was not improved by taking ploidy into account. This was valid for the entire material and for the group with radically removed tumours. Survival analyses were not carried out for the different Dukes' stages in combination with ploidy status because of the small number of observations in the respective groups. The exception was observed for patients with diploid tumours showing very long $T_s$ (highest quartile used as cut-off level). These patients had a higher mortality rate than those with shorter $T_s$-values ($P < 0.001$ for both the entire material and radically removed tumours). No significant difference in survival was observed for $T_s$ when studying the group of aneuploid tumours.

### Clinicopathological parameters

With the exception of sex, topography and ploidy, none of the clinicopathological parameters covaried with parameters associated with proliferation (Table 1). LI/IHC at both the invasive margin and the luminal border showed significant differences between the sexes; where women had higher LIs than men ($P = 0.02$ and $P = 0.02$ respectively). Colorectal cancers in female patients had significantly lower values of $T_{pot}$/IHC at the invasive margin compared with colorectal cancers in men ($P = 0.049$). LI/FCM was also significantly lower in tumours derived from the right colon compared with the left colon and rectum. In contrast, LI/IHC did not differ according to topography for the invasive margin or for the luminal border.

### DISCUSSION

The morphometric procedure used in this study is well known and has been evaluated theoretically (Weibel, 1979). The technique basically assumes a random, systematic sampling procedure and focuses on counting a defined number of nuclei to provide a reliable mean value from each tumour (Gundersen et al, 1988; West and Gundersen, 1990). The comparative test performed in our laboratory, in which our standard non-random procedure of sampling was compared with an optimal random systematic

© Cancer Research Campaign 1998

sampling, gave an excellent correlation, indicating that the results obtained in this study were not significantly influenced by the sampling procedure used. The definition of tumour compartments corresponding to the invasive margin and to the luminal border was done for practical reasons to standardize the morphometric procedure.

Presence of heterogeneity is well known for many solid tumours, including colorectal carcinomas. In this respect, the most extensive studies concern DNA analyses, i.e. evaluation of ploidy (Hiddemann et al, 1986; Scott et al, 1987) and S-phase fraction (Quirke et al, 1985; Lindmark et al, 1991), but also to other proliferative parameters such as PCNA (Teixeira et al, 1994) and expression of Ki-67 (Shepherd et al, 1988). A few reports exist on the presence of intratumoral heterogeneity with respect to LI/IHC and $T_{pot}$/IHC in renal cell carcinoma (Larsson et al, 1994) and to $T_{pot}$/FCM in colorectal cancer (Wilson et al, 1993b). Our study describes the existence of a systematic heterogeneity within colorectal cancer with more pronounced LI at the luminal border compared with the invasive margin for a majority of colorectal cancers. Such a proliferative difference in colorectal cancer is supported by reports after in vitro labelling (LI/IHC) with BrdUrd (Taniyama et al, 1993) and by distribution of Ki-67 tumour cell LI combined with endothelial cell proliferative heterogeneity (Vermeulen et al, 1995).

Evaluation of proliferation with FCM after in vivo incorporation of IdUrd does not in practice allow the different defined tumour compartments to be analysed separately. In addition, it is difficult to distinguish between tumour and non-tumour cells, at least in diploid tumours, because of the admixture of normal, less proliferative cells (Wilson et al, 1991). The more reliable measurements of LI with IHC suggest that LI at the invasive margin is, in comparison with LI/FCM, rather the reverse, with higher LI in diploid than in aneuploid tumours. In contrast, when analysing the LI/IHC at the luminal border, no difference between diploid and aneuploid tumours was observed.

Speculatively, there are two possible major explanations for the difference in proliferation between the luminal border and the invasive margin, although these explanations are not mutually exclusive.

On the one hand, feacal content is known to stimulate cell proliferation and, therefore it seems reasonable that the proliferation rate would be higher at the luminal border and that the proliferative activity would decrease when the diffusion distance from the feacal factors increases. Such proliferative stimulators are, for example, secondary bile acids, mainly lithocholic acid, other steroid-derived compounds, short-chain fatty acids and the direct influence from the bacterial content (Mullan et al, 1990). However, local non-specific regenerative factors from the ulcerative process at the luminal border can also increase the luminal proliferative activity.

On the other hand, tumour cells growing at the invasive margin might be more influenced by factors produced by the surrounding stromal cells rather than by cells at the luminal border. Such an influence includes differences in local levels of growth factors, e.g. transforming growth factor beta (TGF-β). TGF-β has several diverse fields of action in normal cells, eg. decreasing proliferation, increasing differentiation, increasing apoptosis and stimulating angiogenesis (Lawrence et al, 1996). The correlation between decreased proliferation and a higher mortality rate observed in this study could be in accordance with some of the effects of TGF-β. The total action of TGF-β is, however, highly complex and not completely understood. Other factors affecting proliferation of tumour cells are also released from the stromal cells and some of them are known to interfere with the cell cycle. This suggests a possible mechanism for the decreased proliferative activity at the invasive margin.

Tumour cells with invading behaviour require high rates of protein synthesis but are not dependent on DNA synthesis and proliferation (Thorgeirsson et al, 1984). In addition, in malignant gliomas, it is believed that tumour cells must cease proliferation to be able to invade the surrounding tissue (Pilkington et al, 1992). The extracellular matrix proteins seem to inhibit the proliferation of glioma cells and instead stimulate them to migrate (Koochekpour et al, 1995). In addition to these two possible explanations, the presence of separate cell clones at the luminal border and at the invasive margin, and problems of nutrient and oxygen delivery to the invasive margin cannot be excluded.

The systematic heterogeneity observed in this study has implications for sampling when cell kinetic parameters are to be evaluated. As LIs at the luminal border and invasive margin are well correlated, cell kinetic evaluation of samples taken regularly from either of these compartments seems rational. Analyses of randomly extracted samples from colorectal cancers may, however, be of less value.

The prognostic impact of cell kinetic data in colorectal cancer remains questionable. Concerning S-phase fraction, results both with (Bauer et al, 1987; Witzig et al, 1991) and without (Enker et al, 1991) significant prognostic impact have been published. Therefore, according to the consensus by Bauer et al (1993) doubts remain as to whether the S-phase fraction can contribute to the prediction of outcome for patients with colorectal cancer. Cell kinetic studies of colorectal carcinoma using other cell cycle associated parameters are sparse. Rew et al (1993) studied colorectal cancer after in vivo incorporation of BrdUrd and did not find any prognostic impact of flow cytometric evaluation of $T_{pot}$. Morphometric analysis of Ki-67 after immunohistochemical staining of routinely fixed, paraffin-embedded tumour samples (Kubota et al, 1992; Baretton et al, 1996) also failed to predict the outcome.

In contrast to previous reports, our data indicate a negative influence from cell proliferation after IdUrd incorporation in vivo, significant for LI/IHC and $T_{pot}$/IHC in the unselected group of colorectal cancers. One conceivable explanation for the observed impact on prognosis could be the morphometric control of the systematic heterogeneity that exists between the luminal compartment and the invasive margin in our study (extensively discussed by Rew et al (1993). The negative prognostic impact from cell proliferation after in vivo incorporation of IdUrd observed in this study, i.e. shorter survival for colorectal cancer with a low LI or long $T_{pot}$ compared with tumours with high LI and short $T_{pot}$, has to the best of our knowledge not been reported earlier. However, morphometrical evaluation of PCNA in a group of advanced colorectal cancers also showed a correlation between low PCNA LI/IHC and poorer prognosis (Paradiso et al, 1996). Decreased cell proliferation corresponding to lower LI/IHC after in vitro incorporation of BrdUrd in diploid colorectal cancers has previously been correlated with increased numbers of regional lymph node metastases (Taniyama et al, 1993). Furthermore, low LI/IHC for PCNA and Ki-67 correlate to areas with low differentiation at the invasive margin of colorectal cancer (Taniyama et al, 1996). A recent study of cell proliferation in breast cancer also indicates a connection between positive survival outcomes after treatment and high LI/IHC after BrdUrd incorporation (Gamel et al, 1995).

© Cancer Research Campaign 1998

This study provides new insight into the biological behaviour of colorectal cancer. Therefore, additional studies with IdUrd/BrdUrd and other cell cycle-associated parameters, such as Ki-67, are important, provided that the problem of heterogeneity is taken into account.

### ACKNOWLEDGEMENT

This study was supported by grants from the Swedish Cancer Society project no. 2520-B94-08XAC, from the Lion's Cancer Research Foundation, Umeå, and from the Medical Faculty of the University of Umeå. We gratefully acknowledge Professor Hans Jørgen Gundersen, University of Aarhus, for invaluable support in morphometrical competence. We also thank Mrs Ulla-Stina Spetz and Mrs Kerstin Näslund for excellent support in the flow cytometric and histopathological assessment, Mr Björn Tavelin for statistical support and Dr Göran Heinius, District Hospital of Örnsköldsvik, Dr Stefan Spinnell District Hospital of Piteå, Dr Stefan Dedorsson, District Hospital of Luleå and Dr Tommy Löfdahl, District Hospital of Lycksele, for valuable collaboration.

### REFERENCES

Al-Sheneber IF, Shibata HR, Sampalis J and Jothy S (1993) Prognostic significance of proliferating cell nuclear antigen expression in colorectal cancer. *Cancer* **71**: 1954–1959

Baretton GB, Diebold J, Christoforis G, Vogt M, Muller C, Dopfer K, Schneiderbanger K, Schmidt M and Lohrs U (1996) Apoptosis and immunohistochemical bcl-2 expression in colorectal adenomas and carcinomas – aspects of carcinogenesis and prognostic significance. *Cancer* **77**: 255–264

Bauer KD, Lincoln ST, Vera Roman JM, Wallemark CB, Chmiel JS, Madurski ML, Murad T and Scarpelli DG (1987) Prognostic implications of proliferative activity and DNA aneuploidy in colonic adenocarcinomas. *Lab Invest* **57**: 329–335

Bauer KD, Bagwell CB, Giaretti W, Melamed M, Zarbo RJ, Witzig TE and Rabinovitch PS (1993) Consensus review of the clinical utility of DNA flow cytometry in colorectal cancer. *Cytometry* **14**: 486–491

Begg AC, McNally NJ, Shrieve DC and Karcher H (1985) A method to measure the duration of DNA synthesis and the potential doubling time from a single sample. *Cytometry* **6**: 620–626

Bennett MH, Wilson GD, Dische S, Saunders MI, Martindale CA, Robinson BM, O'Halloran AE, Leslie MD and Laing JH (1992) Tumour proliferation assessed by combined histological and flow cytometric analysis: implications for therapy in squamous cell carcinoma in the head and neck. *Br J Cancer* **65**: 870–878

Dische S and Saunders MI (1989) Continuous, hyperfractionated, accelerated radiotherapy (CHART). *Br J Cancer* **59**: 325–326

Enker WE, Kimmel M, Cibas ES, Cranor ML and Melamed MR (1991) DNA/RNA content and proliferative fractions of colorectal carcinomas: a five-year prospective study relating flow cytometry to survival. *J Natl Cancer Inst* **83**: 701–707

Gamel JW, Meyer JS and Province MA (1995) Proliferative rate by S-phase measurement may affect cure of breast carcinoma. *Cancer* **76**: 1009–1018

Gundersen HJG (1977) Notes on the estimation of the numerical density of arbitrary profiles: the edge effect. *J Microsc* **111**: 219–223

Gundersen HJG and Osterby R (1981) Optimizing sampling efficiency of stereological studies in biology: or 'Do more less well!'. *J Microsc* **121**: 65–73

Gundersen HJG and Jensen EB (1987) The efficiency of systematic sampling in stereology and its prediction. *J Microsc* **147**: 229–263

Gundersen HJG, Bendtsen TF, Korbo L, Marcussen N, Moller A, Nielsen K, Nyengaard JR, Pakkenberg B, Sorensen FB, Vesterby A and West MJ (1988) Some new, simple and efficient stereological methods and their use in pathological research and diagnosis. *APMIS* **96**: 379–394

Harlow SP, Eriksen BL, Poggensee L, Chmiel JS, Scarpelli DG, Murad T and Bauer KD (1991) Prognostic implications of proliferative activity and DNA aneuploidy in Astler-Coller Dukes stage C colonic adenocarcinomas. *Cancer Res* **51**: 2403–2409

Hiddemann W, Von Bassewitz DB, Kleinemeier HJ, Schulte Brochterbeck E, Hauss J, Lingemann B, Buchner T and Grundmann E (1986) DNA stemline heterogeneity in colorectal cancer. *Cancer* **58**: 258–263

Holm T, Cedermark B and Rutqvist LE (1994) Local recurrence of rectal adenocarcinoma after 'curative' surgery with and without preoperative radiotherapy. *Br J Surg* **81**: 452–455

International Multicentre Pooled Analysis of Colon Cancer Trials (IMPACT) Investigators (1995) Efficacy of adjuvant fluorouracil and folinic acid in colon cancer. *Lancet* **345**: 939–944

Jass JR, Atkin WS, Cuzick J, Bussey HJ, Morson BC, Northover JM and Todd IP (1986) The grading of rectal cancer: historical perspectives and a multivariate analysis of 447 cases. *Histopathology* **10**: 437–459

Koha M, Caspersson TO, Wikstrom B and Brismar B (1990) Heterogeneity of DNA distribution pattern in colorectal carcinoma. A microspectrophotometric study of fine needle aspirates. *Anal Quant Cytol Histol* **12**: 348–351

Koochekpour S, Merzak A and Pilkington GJ (1995) Extracellular matrix proteins inhibit proliferation, upregulate migration and induce morphological changes in human glioma cell lines. *Eur J Cancer* **31**: 375–380

Kubota Y, Petras RE, Easley KA, Bauer TW, Tubbs RR and Fazio VW (1992) Ki-67-determined growth fraction versus standard staging and grading parameters in colorectal carcinoma. A multivariate analysis. *Cancer* **70**: 2602–2609

Larsson P, Roos G, Stenling R and Ljungberg B (1994) Proliferation of human renal cell carcinoma studied with in vivo iododeoxyuridine labelling and immunohistochemistry. *Scand J Urol Nephrol* **28**: 135–140

Lawrence DA (1996) Transforming growth factor-beta: a general review. *Eur Cytokine Netw* **7**: 363–374

Lindmark G, Glimelius B, Pahlman L and Enblad P (1991) Heterogeneity in ploidy and S-phase fraction in colorectal adenocarcinomas. *Int J Colorectal Dis* **6**: 115–120

Lochrin CA, Wilson GD, McNally NJ, Dische S and Saunders MI (1992) Tumor cell kinetics, local tumor control, and accelerated radiotherapy: a preliminary report. *Int J Radiat Oncol Biol Phys* **24**: 87–91

Macfarlane JK, Ryall RD and Heald RJ (1993) Mesorectal excision for rectal cancer. *Lancet* **341**: 457–460

Moertel CG, Fleming TR, Macdonald JS, Haller DG, Laurie JA, Tangen CM, Ungerleider JS, Emerson WA, Tormey DC, Glick JH, Veeder MH and Maillard JA (1995) Fluorouracil plus levamisole as effective adjuvant therapy after resection of stage III colon carcinoma: a final report. *Ann Intern Med* **122**: 321–326

Mullan FJ, Wilson HK, Majury CW, Mills JO, Cromie AJ, Campbell GR and McKelvey ST (1990) Bile acids and the increased risk of colorectal tumours after truncal vagotomy. *Br J Surgery* **77**: 1085–1090

Nylander K, Anneroth G, Gustafsson H, Roos G, Stenling R and Zackrisson B (1994) Cell kinetics of head and neck squamous cell carcinomas. Prognostic implications. *Acta Oncol* **33**: 23–28

Paradiso A, Rabinovich M, Vallejo C, Machiavelli M, Romero A, Perez J, Lacava J, Cuevas MA, Rodriquez R, Leone B, Sapia MG, Simone G and De Lena M (1996) p53 and PCNA expression in advanced colorectal cancer: Response to chemotherapy and long-term prognosis. *Int J Cancer* **69**: 437–441

Pilkington GJ (1992) Glioma heterogeneity in vitro: the significance of growth factors and gangliosides. *Neuropathol Appl Neurobiol* **18**: 434–442

Quirke P, Dyson JED, Dixon MF, Bird CC and Joslin CAF (1985) Heterogeneity of colorectal adenocarcinomas evaluated by flow cytometry and histopathology. *Br J Cancer* **51**: 99–106

Rew DA (1993) Cell proliferation, tumour growth and clinical outcome: gains and losses in intestinal cancer. *Ann R Coll Surg Engl* **75**: 397–404

Rew DA, Wilson GD, Taylor I and Weaver PC (1991) Proliferation characteristics of human colorectal carcinomas measured in vivo. *Br J Surg* **78**: 60–66

Riccardi A, Giordano M, Danova M, Girino M, Brugnatelli S, Ucci G and Mazzini G (1991) Cell kinetics with in vivo bromodeoxyuridine and flow cytometry: clinical significance in acute non-lymphoblastic leukaemia. *Eur J Cancer* **27**: 882–887

Riethmuller G, Schneider Gadicke E, Schlimok G, Schmiegel W, Raab R, Hoffken K, Gruber R, Pichlmaier H, Hirche H, Pichlmayr R, Buggish P, Witte J and The German Cancer Aid 17-1A Study Group (1994) Randomised trial of monoclonal antibody for adjuvant therapy of resected Dukes' C colorectal carcinoma. *Lancet* **343**: 1177–1183

Scott NA, Grande JP, Weiland LH, Pemberton JH, Beart Jr RW and Lieber MM (1987) Flow cytometric DNA patterns from colorectal cancers – how reproducible are they? *Mayo Clin Proc* **62**: 331–337

Shepherd NA, Richman PI and England J (1988) Ki-67 derived proliferative activity in colorectal adenocarcinoma with prognostic correlations. *J Pathol* **155**: 213–219

© Cancer Research Campaign 1998

Steel GG (1977) *Growth kinetics of tumours*. Clarendon Press: Oxford.

Taniyama K, Suzuki H, Matsumoto M, Hakamada K, Toyama K and Tahara E (1993) Relationships between nodal status and cell kinetics, DNA ploidy pattern and histopathology of the deeply infiltrating sites in colorectal adenocarcinoma. *Acta Pathol Jpn* **43**: 590–596

Taniyama K, Sasaki N, Wada S, Sasaki M, Miyoshi N, Nakai H, Kodama S, Nakatsuka H and Tahara E (1996) Comparison of proliferative activities and metastases between two subtypes classified at the deeply infiltrating sites of colorectal moderately differentiated adenocarcinomas. *Pathology International* **46**: 195–203

Teixeira CR, Tanaka S, Haruma K, Yoshihara M, Sumii K and Kajiyama G (1994) Proliferating cell nuclear antigen expression at the invasive tumor margin predicts malignant potential of colorectal carcinomas. *Cancer* **73**: 575–579

Terry NH, Meistrich ML, Roubein LD, Lynch PM, Dubrow RA and Rich TA (1995) Cellular kinetics in rectal cancer. *Br J Cancer* **72**: 435–441

Thorgeirsson UP, Turpeenniemi Hujanen T, Neckers LM, Johnson DW and Liotta LA (1984) Protein synthesis but not DNA synthesis is required for tumor cell invasion in vitro. *Invasion Metastasis* **4**: 73–83

Vermeulen PB, Verhoeven D, Hubens G, Van Marck E, Goovaerts G, Huyghe M, De Bruijn EA, Van Oosterom AT and Dirix LY (1995) Microvessel density, endothelial cell proliferation and tumour cell proliferation in human colorectal adenocarcinomas. *Ann Oncol* **6**: 59–64

Weibel ER (1979) *Practical methods for biological morphometry*. Academic Press: London

West MJ and Gundersen HJ (1990) Unbiased stereological estimation of the number of neurons in the human hippcampus. *J Comp Neurol* **296**: 1–22

Wilson GD (1991) Assessment of human tumour proliferation using bromodeoxyuridine – current status. *Acta Oncol* **30**: 903–910

Wilson GD and McNally MC (1992) Measurement of cell proliferation using bromodeoxyuridine. In *Assessment of Cell Proliferation in Clinical Practice*, Hall PA. (ed.), pp. 113–139. Springer: London

Wilson MS, West CM, Wilson GD, Roberts SA, James RD and Schofield PF (1993*a*) An assessment of the reliability and reproducibility of measurement of potential doubling times ($T_{pot}$) in human colorectal cancers. *Br J Cancer* **67**: 754–759

Wilson MS, West CM, Wilson GD, Roberts SA, James RD and Schofield PF (1993*b*) Intra-tumoral heterogeneity of tumour potential doubling times ($T_{pot}$) in colorectal cancer. *Br J Cancer* **68**: 501–506

Witzig TE, Loprinzi CL, Gonchoroff NJ, Reiman HM, Cha SS, Wieand HS, Katzmann JA, Paulsen JK and Moertel CG (1991) DNA ploidy and cell kinetic measurements as predictors of recurrence and survival in stages B2 and C colorectal adenocarcinoma. *Cancer* **68**: 879–888

© Cancer Research Campaign 1998


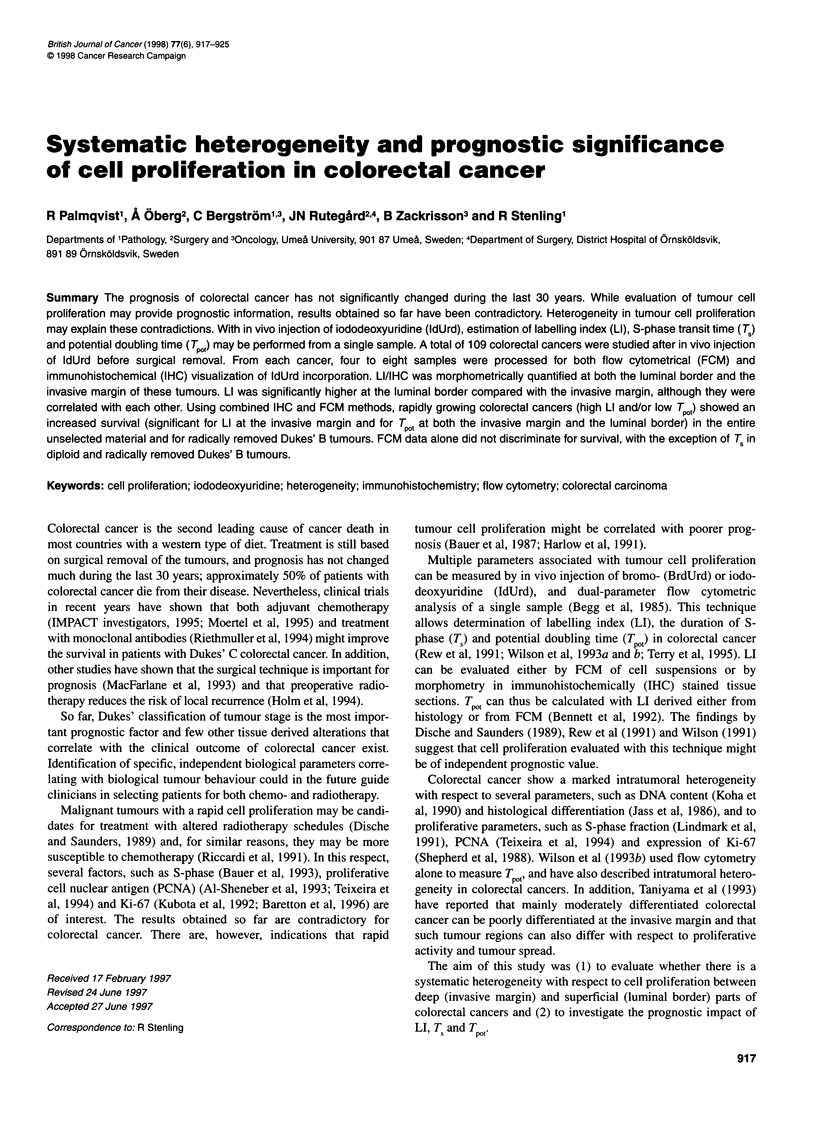

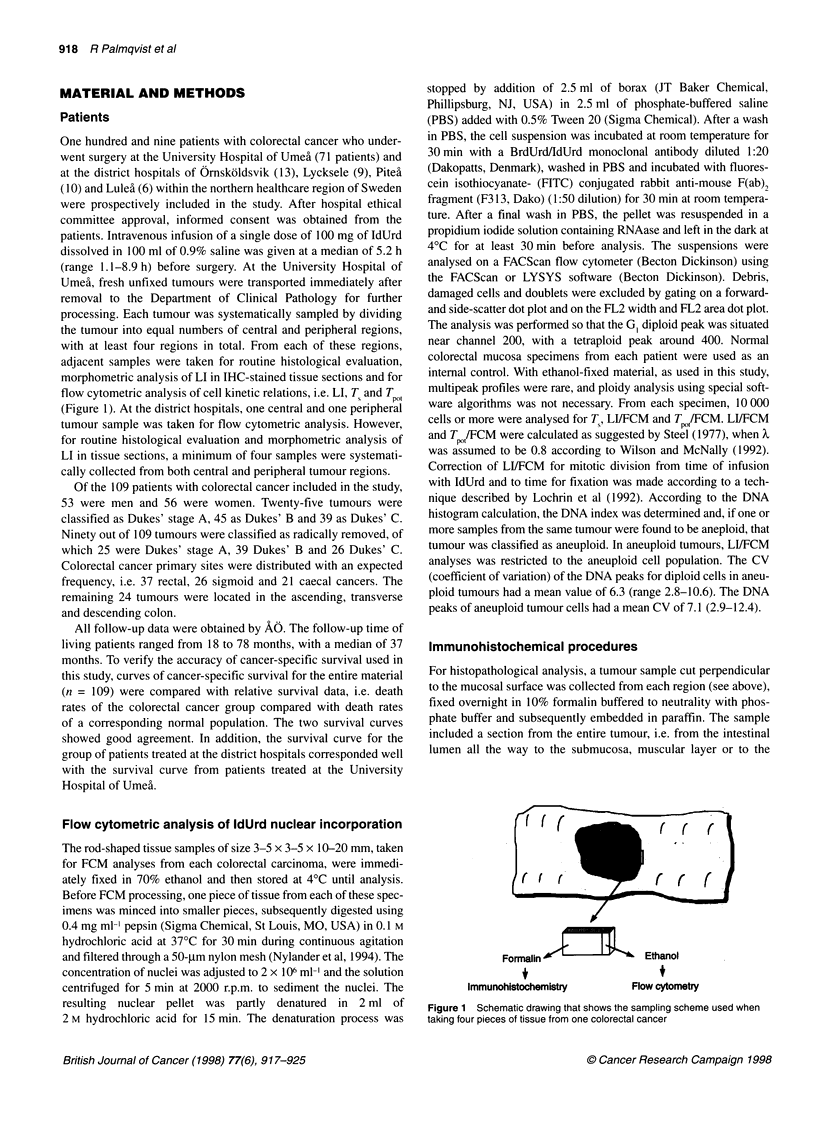

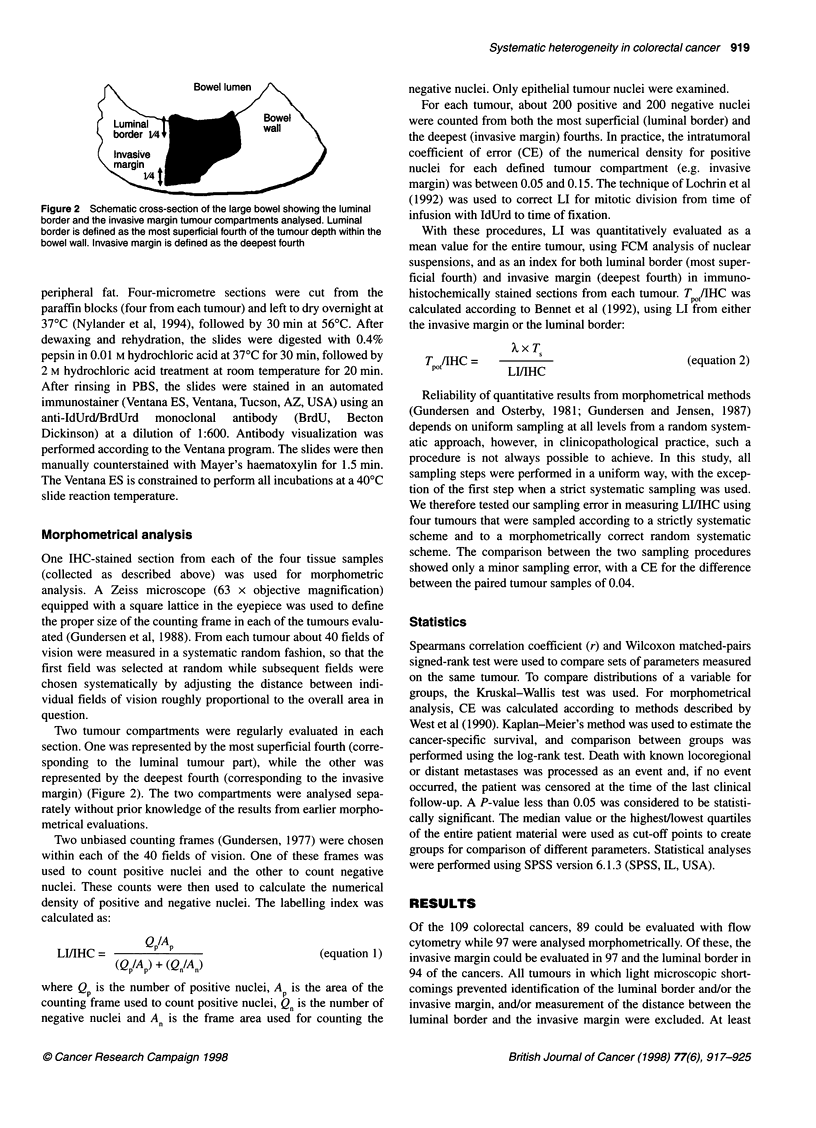

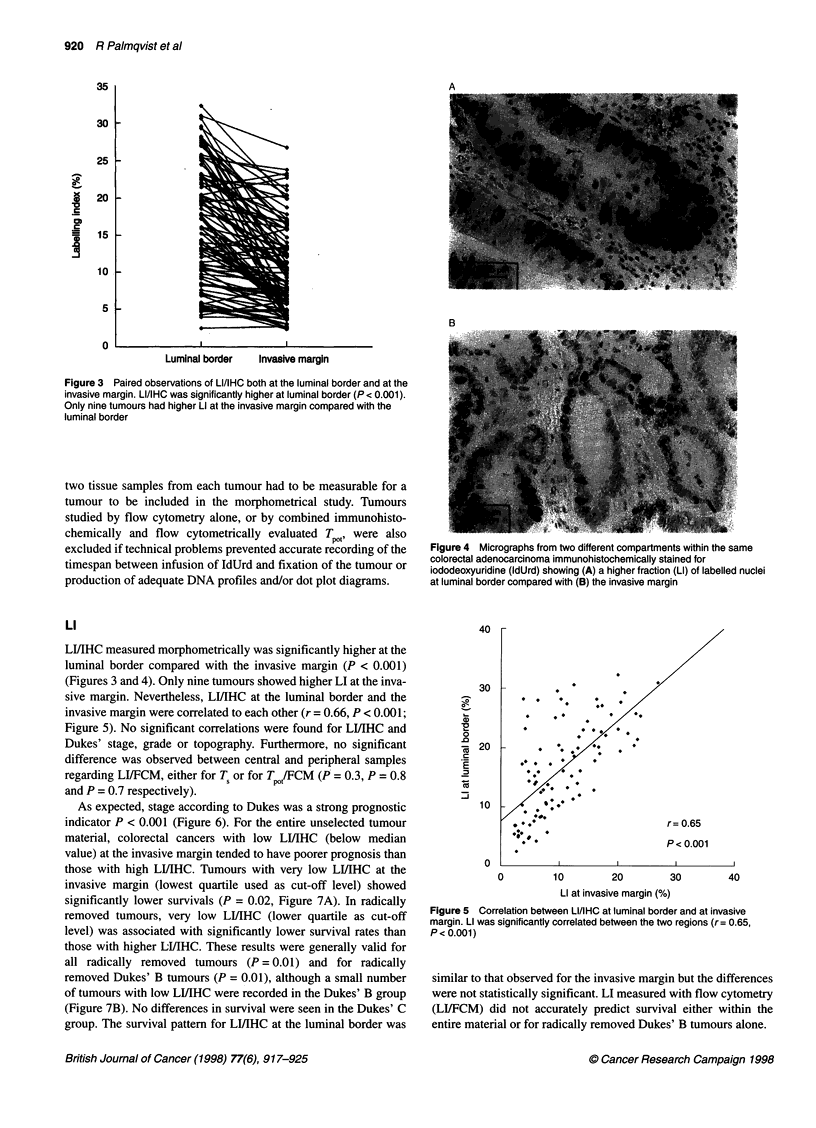

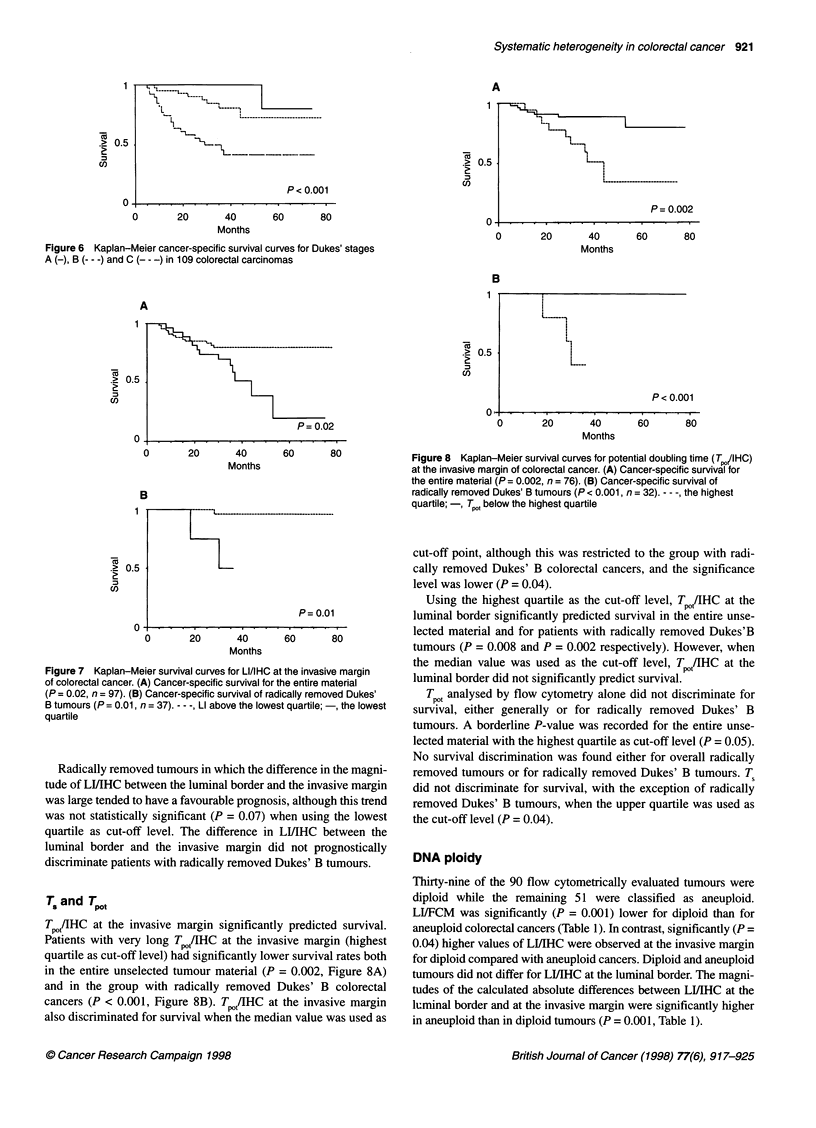

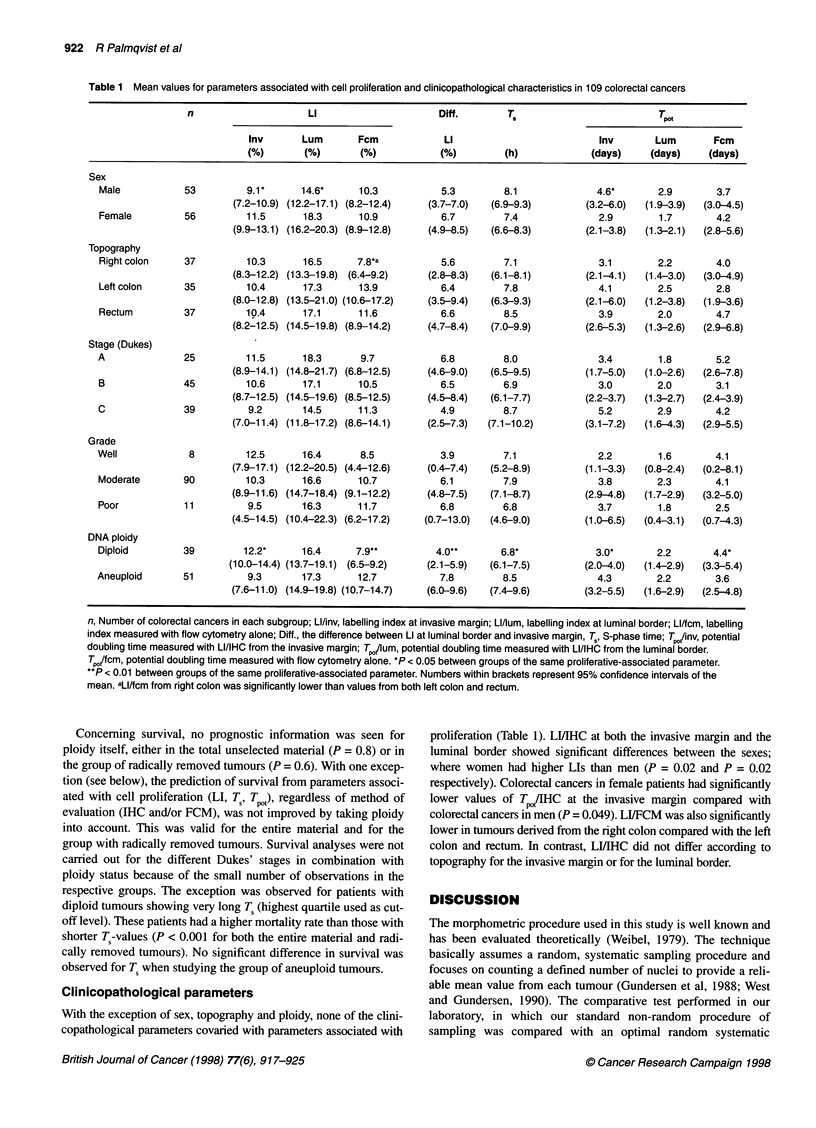

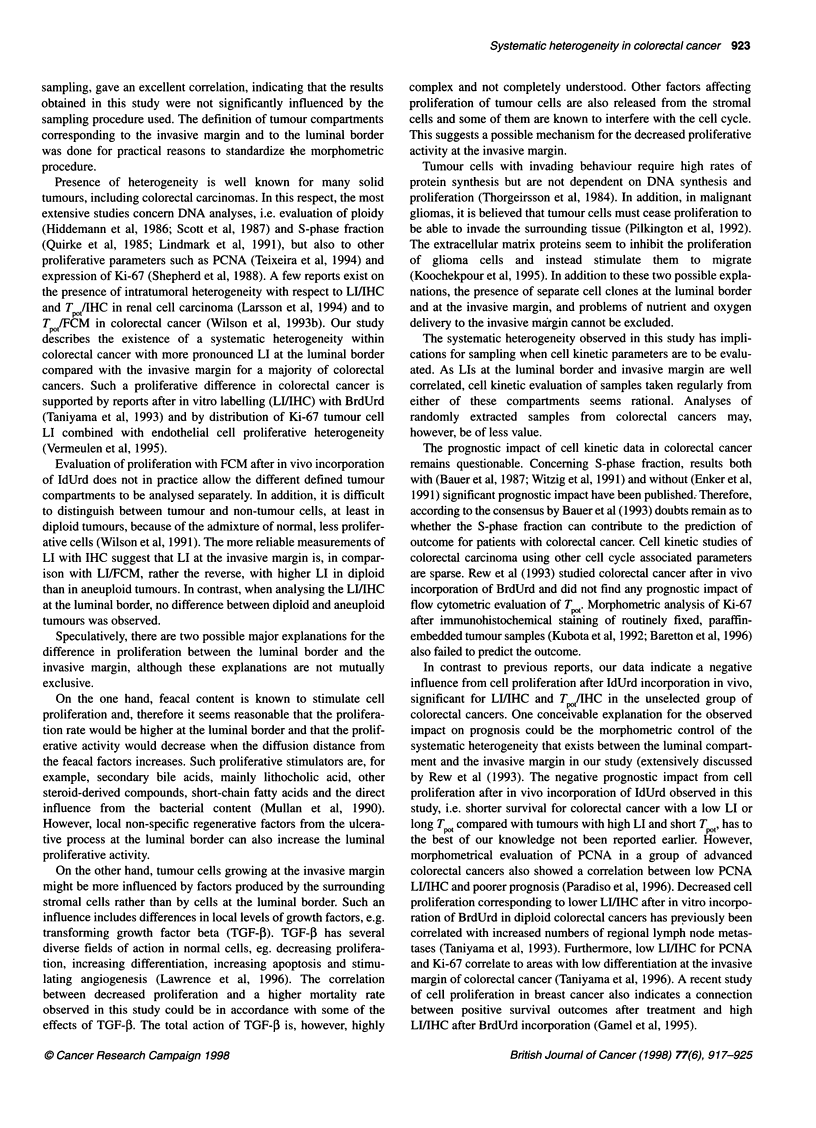

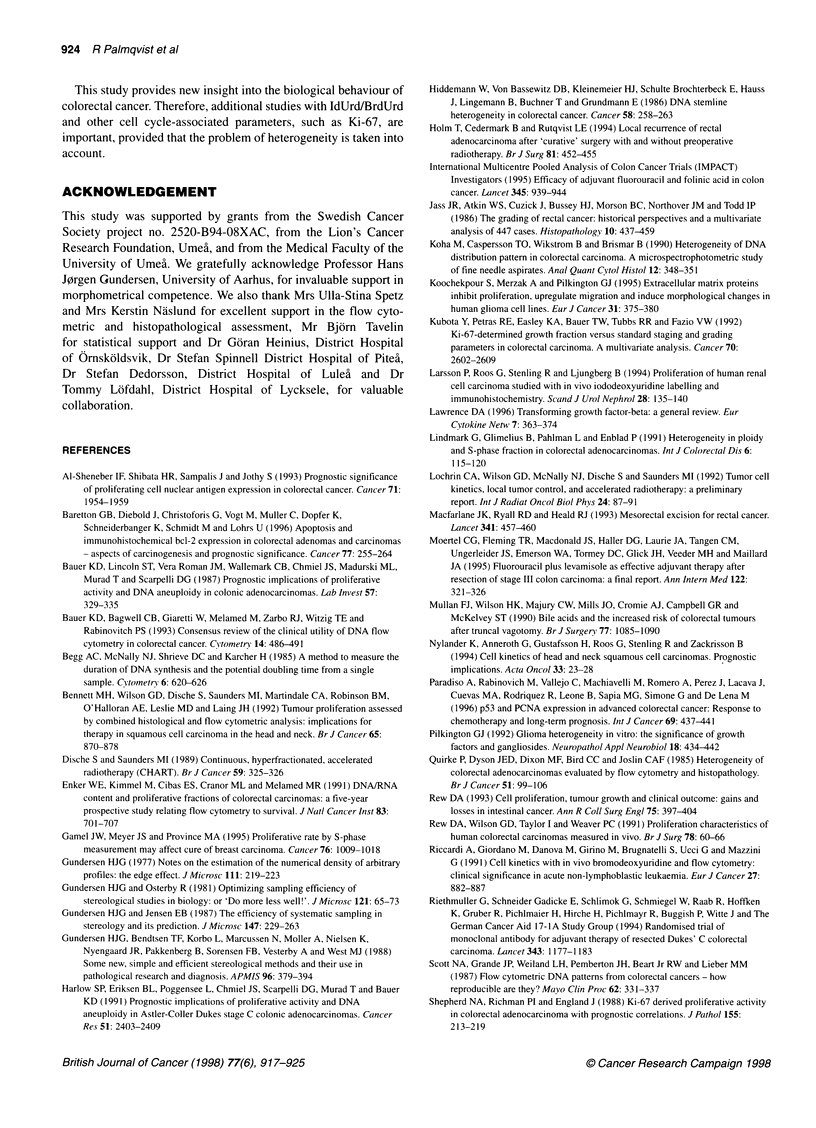

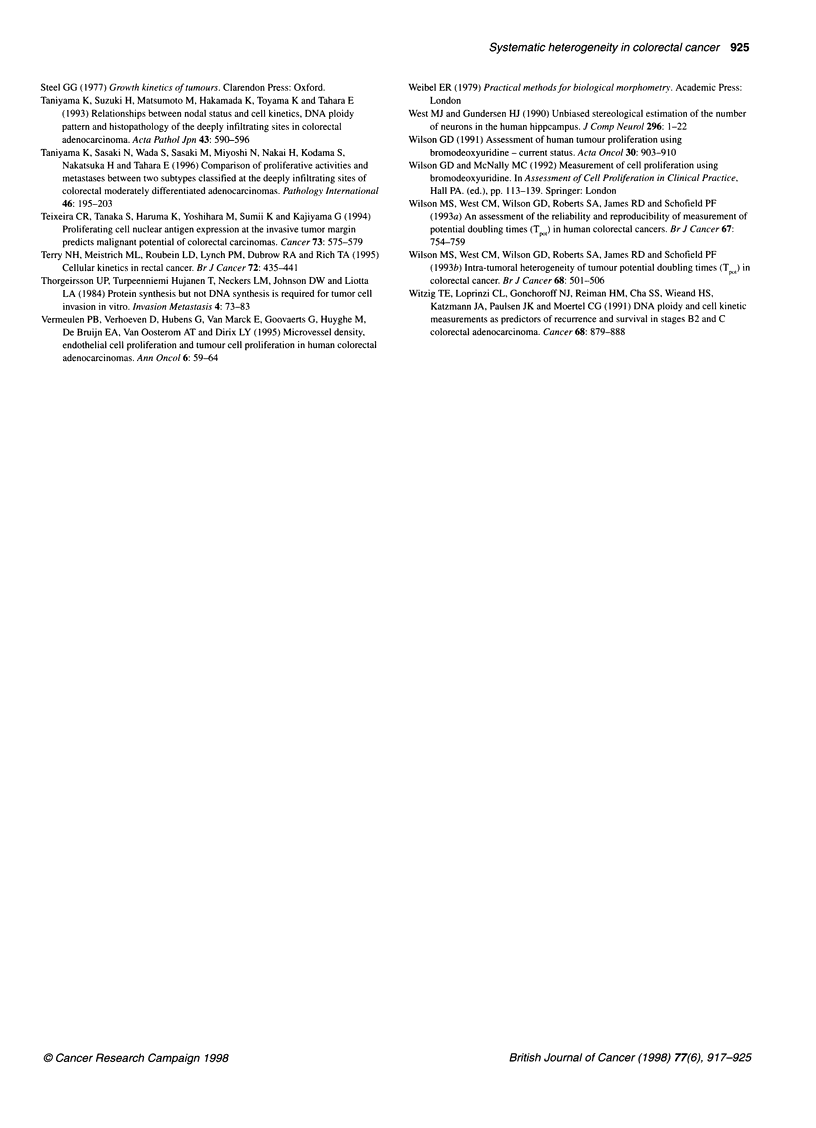


## References

[OCR_00959] Baretton G. B., Diebold J., Christoforis G., Vogt M., Müller C., Dopfer K., Schneiderbanger K., Schmidt M., Löhrs U. (1996). Apoptosis and immunohistochemical bcl-2 expression in colorectal adenomas and carcinomas. Aspects of carcinogenesis and prognostic significance.. Cancer.

[OCR_00972] Bauer K. D., Bagwell C. B., Giaretti W., Melamed M., Zarbo R. J., Witzig T. E., Rabinovitch P. S. (1993). Consensus review of the clinical utility of DNA flow cytometry in colorectal cancer.. Cytometry.

[OCR_00966] Bauer K. D., Lincoln S. T., Vera-Roman J. M., Wallemark C. B., Chmiel J. S., Madurski M. L., Murad T., Scarpelli D. G. (1987). Prognostic implications of proliferative activity and DNA aneuploidy in colonic adenocarcinomas.. Lab Invest.

[OCR_00977] Begg A. C., McNally N. J., Shrieve D. C., Kärcher H. (1985). A method to measure the duration of DNA synthesis and the potential doubling time from a single sample.. Cytometry.

[OCR_00982] Bennett M. H., Wilson G. D., Dische S., Saunders M. I., Martindale C. A., Robinson B. M., O'Halloran A. E., Leslie M. D., Laing J. H. (1992). Tumour proliferation assessed by combined histological and flow cytometric analysis: implications for therapy in squamous cell carcinoma in the head and neck.. Br J Cancer.

[OCR_00990] Dische S., Saunders M. I. (1989). Continuous, hyperfractionated, accelerated radiotherapy (CHART).. Br J Cancer.

[OCR_00994] Enker W. E., Kimmel M., Cibas E. S., Cranor M. L., Melamed M. R. (1991). DNA/RNA content and proliferative fractions of colorectal carcinomas: a five-year prospective study relating flow cytometry to survival.. J Natl Cancer Inst.

[OCR_01001] Gamel J. W., Meyer J. S., Province M. A. (1995). Proliferative rate by S-phase measurement may affect cure of breast carcinoma.. Cancer.

[OCR_01016] Gundersen H. J., Bendtsen T. F., Korbo L., Marcussen N., Møller A., Nielsen K., Nyengaard J. R., Pakkenberg B., Sørensen F. B., Vesterby A. (1988). Some new, simple and efficient stereological methods and their use in pathological research and diagnosis.. APMIS.

[OCR_01012] Gundersen H. J., Jensen E. B. (1987). The efficiency of systematic sampling in stereology and its prediction.. J Microsc.

[OCR_01009] Gundersen H. J., Osterby R. (1981). Optimizing sampling efficiency of stereological studies in biology: or 'do more less well!'.. J Microsc.

[OCR_01022] Harlow S. P., Eriksen B. L., Poggensee L., Chmiel J. S., Scarpelli D. G., Murad T., Bauer K. D. (1991). Prognostic implications of proliferative activity and DNA aneuploidy in Astler-Coller Dukes stage C colonic adenocarcinomas.. Cancer Res.

[OCR_01029] Hiddemann W., Von Bassewitz D. B., Kleinemeier H. J., Schulte-Brochterbeck E., Hauss J., Lingemann B., Büchner T., Grundmann E. (1986). DNA stemline heterogeneity in colorectal cancer.. Cancer.

[OCR_01034] Holm T., Cedermark B., Rutqvist L. E. (1994). Local recurrence of rectal adenocarcinoma after 'curative' surgery with and without preoperative radiotherapy.. Br J Surg.

[OCR_01044] Jass J. R., Atkin W. S., Cuzick J., Bussey H. J., Morson B. C., Northover J. M., Todd I. P. (1986). The grading of rectal cancer: historical perspectives and a multivariate analysis of 447 cases.. Histopathology.

[OCR_01049] Koha M., Caspersson T. O., Wikström B., Brismar B. (1990). Heterogeneity of DNA distribution pattern in colorectal carcinoma. A microspectrophotometric study of fine needle aspirates.. Anal Quant Cytol Histol.

[OCR_01054] Koochekpour S., Merzak A., Pilkington G. J. (1995). Extracellular matrix proteins inhibit proliferation, upregulate migration and induce morphological changes in human glioma cell lines.. Eur J Cancer.

[OCR_01059] Kubota Y., Petras R. E., Easley K. A., Bauer T. W., Tubbs R. R., Fazio V. W. (1992). Ki-67-determined growth fraction versus standard staging and grading parameters in colorectal carcinoma. A multivariate analysis.. Cancer.

[OCR_01066] Larsson P., Roos G., Stenling R., Ljungberg B. (1994). Proliferation of human renal cell carcinoma studied with in vivo iododeoxyuridine labelling and immunohistochemistry.. Scand J Urol Nephrol.

[OCR_01071] Lawrence D. A. (1996). Transforming growth factor-beta: a general review.. Eur Cytokine Netw.

[OCR_01075] Lindmark G., Glimelius B., Påhlman L., Enblad P. (1991). Heterogeneity in ploidy and S-phase fraction in colorectal adenocarcinomas.. Int J Colorectal Dis.

[OCR_01080] Lochrin C. A., Wilson G. D., McNally N. J., Dische S., Saunders M. I. (1992). Tumor cell kinetics, local tumor control, and accelerated radiotherapy: a preliminary report.. Int J Radiat Oncol Biol Phys.

[OCR_01085] MacFarlane J. K., Ryall R. D., Heald R. J. (1993). Mesorectal excision for rectal cancer.. Lancet.

[OCR_01089] Moertel C. G., Fleming T. R., Macdonald J. S., Haller D. G., Laurie J. A., Tangen C. M., Ungerleider J. S., Emerson W. A., Tormey D. C., Glick J. H. (1995). Fluorouracil plus levamisole as effective adjuvant therapy after resection of stage III colon carcinoma: a final report.. Ann Intern Med.

[OCR_01096] Mullan F. J., Wilson H. K., Majury C. W., Mills J. O., Cromie A. J., Campbell G. R., McKelvey S. T. (1990). Bile acids and the increased risk of colorectal tumours after truncal vagotomy.. Br J Surg.

[OCR_01101] Nylander K., Anneroth G., Gustafsson H., Roos G., Stenling R., Zackrisson B. (1994). Cell kinetics of head and neck squamous cell carcinomas. Prognostic implications.. Acta Oncol.

[OCR_01106] Paradiso A., Rabinovich M., Vallejo C., Machiavelli M., Romero A., Perez J., Lacava J., Cuevas M. A., Rodriquez R., Leone B. (1996). p53 and PCNA expression in advanced colorectal cancer: response to chemotherapy and long-term prognosis.. Int J Cancer.

[OCR_01113] Pilkington G. J. (1992). Glioma heterogeneity in vitro: the significance of growth factors and gangliosides.. Neuropathol Appl Neurobiol.

[OCR_01117] Quirke P., Dyson J. E., Dixon M. F., Bird C. C., Joslin C. A. (1985). Heterogeneity of colorectal adenocarcinomas evaluated by flow cytometry and histopathology.. Br J Cancer.

[OCR_01122] Rew D. A. (1993). Cell proliferation, tumour growth and clinical outcome: gains and losses in intestinal cancer.. Ann R Coll Surg Engl.

[OCR_01126] Rew D. A., Wilson G. D., Taylor I., Weaver P. C. (1991). Proliferation characteristics of human colorectal carcinomas measured in vivo.. Br J Surg.

[OCR_01130] Riccardi A., Giordano M., Danova M., Girino M., Brugnatelli S., Ucci G., Mazzini G. (1991). Cell kinetics with in vivo bromodeoxyuridine and flow cytometry: clinical significance in acute non-lymphoblastic leukaemia.. Eur J Cancer.

[OCR_01137] Riethmüller G., Schneider-Gädicke E., Schlimok G., Schmiegel W., Raab R., Höffken K., Gruber R., Pichlmaier H., Hirche H., Pichlmayr R. (1994). Randomised trial of monoclonal antibody for adjuvant therapy of resected Dukes' C colorectal carcinoma. German Cancer Aid 17-1A Study Group.. Lancet.

[OCR_01145] Scott N. A., Grande J. P., Weiland L. H., Pemberton J. H., Beart R. W., Lieber M. M. (1987). Flow cytometric DNA patterns from colorectal cancers--how reproducible are they?. Mayo Clin Proc.

[OCR_01150] Shepherd N. A., Richman P. I., England J. (1988). Ki-67 derived proliferative activity in colorectal adenocarcinoma with prognostic correlations.. J Pathol.

[OCR_01167] Taniyama K., Sasaki N., Wada S., Sasaki M., Miyoshi N., Nakai H., Kodama S., Nakatsuka H., Tahara E. (1996). Comparison of proliferative activities and metastases between two subtypes classified at the deeply infiltrating sites of colorectal moderately differentiated adenocarcinomas.. Pathol Int.

[OCR_01161] Taniyama K., Suzuki H., Matsumoto M., Hakamada K., Toyama K., Tahara E. (1993). Relationships between nodal status and cell kinetics, DNA ploidy pattern and histopathology of the deeply infiltrating sites in colorectal adenocarcinoma.. Acta Pathol Jpn.

[OCR_01175] Teixeira C. R., Tanaka S., Haruma K., Yoshihara M., Sumii K., Kajiyama G. (1994). Proliferating cell nuclear antigen expression at the invasive tumor margin predicts malignant potential of colorectal carcinomas.. Cancer.

[OCR_01181] Terry N. H., Meistrich M. L., Roubein L. D., Lynch P. M., Dubrow R. A., Rich T. A. (1995). Cellular kinetics in rectal cancer.. Br J Cancer.

[OCR_01185] Thorgeirsson U. P., Turpeenniemi-Hujanen T., Neckers L. M., Johnson D. W., Liotta L. A. (1984). Protein synthesis but not DNA synthesis is required for tumor cell invasion in vitro.. Invasion Metastasis.

[OCR_01190] Vermeulen P. B., Verhoeven D., Hubens G., Van Marck E., Goovaerts G., Huyghe M., De Bruijn E. A., Van Oosterom A. T., Dirix L. Y. (1995). Microvessel density, endothelial cell proliferation and tumour cell proliferation in human colorectal adenocarcinomas.. Ann Oncol.

[OCR_01204] Wilson G. D. (1991). Assessment of human tumour proliferation using bromodeoxyuridine--current status.. Acta Oncol.

[OCR_01213] Wilson M. S., West C. M., Wilson G. D., Roberts S. A., James R. D., Schofield P. F. (1993). An assessment of the reliability and reproducibility of measurement of potential doubling times (Tpot) in human colorectal cancers.. Br J Cancer.

[OCR_01219] Wilson M. S., West C. M., Wilson G. D., Roberts S. A., James R. D., Schofield P. F. (1993). Intra-tumoral heterogeneity of tumour potential doubling times (Tpot) in colorectal cancer.. Br J Cancer.

[OCR_01224] Witzig T. E., Loprinzi C. L., Gonchoroff N. J., Reiman H. M., Cha S. S., Wieand H. S., Katzmann J. A., Paulsen J. K., Moertel C. G. (1991). DNA ploidy and cell kinetic measurements as predictors of recurrence and survival in stages B2 and C colorectal adenocarcinoma.. Cancer.

[OCR_00954] al-Sheneber I. F., Shibata H. R., Sampalis J., Jothy S. (1993). Prognostic significance of proliferating cell nuclear antigen expression in colorectal cancer.. Cancer.

